# Influence of Olive Oil and Its Components on Breast Cancer: Molecular Mechanisms

**DOI:** 10.3390/molecules27020477

**Published:** 2022-01-12

**Authors:** Raquel Moral, Eduard Escrich

**Affiliations:** Department of Cell Biology, Physiology and Immunology, Faculty of Medicine, Universitat Autònoma de Barcelona, 08193 Bellaterra, Spain; Eduard.Escrich@uab.cat

**Keywords:** olive oil, EVOO, breast cancer, minor compounds, apoptosis, proliferation, migration

## Abstract

Breast cancer is the most frequent malignant neoplasia and a leading cause of mortality in women worldwide. The Mediterranean diet has been proposed as a healthy dietary pattern with protective effects in several chronic diseases, including breast cancer. This diet is characterized by the consumption of abundant plant foods and olive oil as the principal source of fat, which is considered one of the main components with potential antioxidant, anti-inflammatory and anticancer effects. Extra-virgin olive oil (EVOO) has several bioactive compounds, mainly including monounsaturated fatty acids, triterpenes and polyphenols, such as phenolic alcohols (e.g., hydroxytyrosol), secoiridoids (e.g., oleuropein and oleocanthal), lignans (e.g., pinoresinol) or flavonoids (e.g., luteolin). While epidemiological evidence is still limited, experimental in vivo and in vitro data have shown a protective effect of this oil and its compounds on mammary carcinogenesis. Such effects account through complex and multiple mechanisms, including changes in epigenetics, transcriptome and protein expression that modulate several signaling pathways. Molecular targets of EVOO compounds have a role in the acquisition of cancer hallmarks. Although further research is needed to elucidate their beneficial effects on human prevention and progression of the disease, evidence points to EVOO in the context of the Mediterranean diet as a heathy choice, while EVOO components may be promising adjuvants in anticancer strategies.

## 1. Introduction

Breast cancer has been the most commonly diagnosed cancer in 2020, with high rates of incidence and mortality in women worldwide [1,2]. Geographical differences in incidence rates suggest an effect of environmental and lifestyle factors in the etiology of this neoplasia [1]. Lifestyles have attracted great scientific interest since they are modifiable risk factors for cancer, with healthy diets, body mass index and exercise showing a significant impact on this disease. Thus, different dietary patterns have been associated with the risk of breast cancer and dietary lipids have a key role on such effect [3]. Human studies regarding such association are controversial; however, experimental studies have provided strong data supporting an effect of dietary lipids on breast cancer [4,5]. This potential modulatory influence depends on the total amount and on the specific type of dietary fat. Hence, diets rich in n-6 polyunsaturated fatty acids (PUFA), especially linoleic acid, have shown stimulating effects on mammary cancer in animal models. Saturated fats and trans fatty acids have also shown to enhance carcinogenesis [4,5]. On the contrary, n-3 PUFA (mainly the long chain n-3 eicosapentaenoic and docosahexaenoic acids), but also gamma-linolenic acid and conjugated linoleic acid, has shown inhibitory effects on cancer cells. Diets with high ratios of n-3/n-6 PUFAs have also demonstrated protective effects [4,5]. The influence of n-9 monounsaturated fatty acids (MUFA) still remains unclear and studies have reported from weak-promoting to protective effects on experimental mammary carcinogenesis [4,5]. Olive oil, rich in n-9 MUFA oleic acid, is the main source of fat in the Mediterranean diet. This diet has been proposed as healthy dietary pattern associated to a decreased risk for some chronic diseases such as cardiovascular disease, obesity and cancer. The Mediterranean diet is characterized by abundant use of olive oil; abundant and varied plant foods (fruits, vegetables, cereals, legumes and nuts); moderate intake of red wine with meals; moderate consumption of fish and seafood, dairy products, poultry and eggs; and low consumption of red meat and sweets [6]. The Mediterranean diet is a pattern historically linked to decreased rates of breast cancer in Mediterranean countries. Actually, breast and other cancer mortality rates have been increasing in southern European countries over the years, which has been correlated with changes in dietary patterns, including the decrease in olive oil consumption and the increase in the consumption of seed oils [7].

Olive oil is obtained from the fruit of the olive tree (*Olea europaea* L.) and has a particular chemical composition not only for its lipid profile but also for a significant quantity of minor compounds, some of them highly bioactive. The specific components depend, to a large extent, on the quality of the oil. Virgin olive oils are the oils extracted mechanically from olives and with no other treatments. Afterward, they are divided according to their acidity, with the lowest corresponding to the extra-virgin olive oil (EVOO). Only virgin olive oils preserve their minor compounds. Refined olive oil is obtained from virgin oil by refining methods. What is known simply as “olive oil” is a blend of refined and virgin olive oils [8,9]. In addition to its quality, olive oil’s specific composition depends on many other parameters, including cultivar, growing area, environmental conditions, pedology, harvest time and system, extraction method, or storage conditions [9,10].

Olive oil composition can be divided into two fractions, the major components (representing more than 98% of total oil weight), which is the saponifiable fraction and includes triacylglycerides and derivatives; and the minor unsaponifiable fraction [8,11,12,13,14]. The fatty acid profile composing the major fraction of a typical olive oil is represented by MUFA oleic acid (55–83%) and palmitoleic acid (0.3–3.5%); palmitic (7.5–20%) and stearic (0.5–5%) saturated fatty acids; n-6 PUFA linoleic acid (3.5–21%); n-3 PUFA linolenic acid (0–1%); and little quantities of other fatty acids (myristic, 14:0; margaric, 17:0; margaroleic, 17:1n9; arachidic, 20:0; eicosenoic, 20:1n9; docosanoic, 22:0; lignoceric, 24:0) [11,15,16,17]. The olive oil unsaponifiable fraction (1–2% of total weight) is characterized by chemical variability and complexity and more than 230 components from different chemical classes have been identified. This unsaponifiable fraction includes triterpenic dialcohols and acids (20–200 mg/kg [18,19]); sterols (1000–5000 mg/kg [11,18]); hydrocarbons, such as squalene (1000–8000 mg/kg [11,18]), n-alkanes and alkenes (up to 330 mg/kg [13,20]), or carotenoids (β-carotene is the most abundant, with 1–11 mg/kg [11,21]); pigments (5–30 mg/kg [21]); and phenolic compounds (lipophilic and hydrophilic). The most important lipophilic phenols are tocopherols (the most abundant is α-tocopherol, with 12–400 mg/kg [11]). In relation to hydrophilic phenols (40–1000 mg/kg [11]), several chemical classes have been identified, such as secoiridois, flavonoids or lignans [12]. Other components are also found in the unsaponifiable EVOO fraction, such as aliphatic alcohols, waxes and many volatiles compounds [11,13]. Table 1 shows the composition of typical EVOO, with the main fatty acids found in the saponifiable fraction, as well as the main chemical classes found in the unsaponifiable fraction.

In the last years, much attention has been given to the potential health benefits of olive oil and the effects of different EVOO components [9,14,22]. Virgin olive oil is considered a healthy fat and two health claims for the beneficial effects of its lipid profile and polyphenol content have been announced by the European Food Safety Authority (EFSA) [23,24]. In relation to cancer, several minor components have demonstrated potential antitumor effects [25], especially hydroxytyrosol [26], secoiridoids [26,27,28], flavonoids [29], lignans [30] and triterpenes [31]. Thus, the purpose of this article is to review the effects of olive oil and its components on breast cancer and obtain insight into the potential mechanisms of action associated to these effects.

## 2. Human Data: Epidemiological Studies on the Effects of Mediterranean Diet and Olive Oil on Breast Cancer Risk

Epidemiological studies have been designed in order to associate the risk of breast cancer with the Mediterranean diet, olive oil consumption, or intake of some EVOO components. These human investigations are mainly based on case-control studies with fewer long-prospective and interventional ones and the results are not fully consistent and conclusive. Thus, case-control studies have showed inverse associations between olive oil consumption and the risk of this neoplasm, with decreased risk for the highest consumption of olive oil described in cohorts conducted in Mediterranean countries such as Greece [32], Italy [33], Kuwait [34] and Spain [35,36]. In the multi-center case-control study EURAMIC, oleic acid concentration in fat stores showed an inverse association with breast cancer but only in the Spanish center (odds ratio, 0.40), suggesting the role of other olive oil compounds beyond oleic acid in its potential protective effect [37]. In this sense, the SUN trial has investigated the association with polyphenol consumption, finding an inverse association between total polyphenol intake and breast cancer risk for postmenopausal women. Thus, there was a 69% risk reduction in the third tertile of polyphenol intake (>800 mg/day) in comparison to the first tertile (<566 mg/day) [38].

On the other hand, meta-analyses combine data from independent primary studies with a large number of participants. In general, those meta-analysis including case-control studies have also observed significant associations of olive oil consumption and reduction in the risk of developing breast cancer, reporting up to five-fold differences in subjects consuming olive oil versus those consuming butter and a relative risk of 0.62 for the highest versus the lowest levels of olive oil intake [39,40]. Prospective cohort studies have also evaluated the association between the Mediterranean diet or olive oil and breast cancer risk. The European Prospective Investigation into Cancer and Nutrition (EPIC) study is one of the largest cohort studies designed to investigate the relationships between diet, nutritional status, lifestyle and environmental factors and the incidence of cancer and other chronic diseases. In a study focused on the Mediterranean diet pattern, the EPIC-Greece cohort reported marginally significant inverse association with breast cancer risk among postmenopausal women (22% reduction every 2-point increase in conformity to the Mediterranean diet) [41]. This negative association resulted significant for estrogen receptor-negative (ER−) breast cancer in two studies [42,43], although, in other cohorts, no clear association has been found [44,45,46,47]. Meta-analyses including cohort studies have reported similar results, also finding inverse associations of the Mediterranean diet with breast cancer and mortality [48] and more strongly with ER− breast cancer [43,49]. It is more difficult to establish associations with Mediterranean diet components and fewer studies have addressed the influence of olive oil consumption. In this sense, a study with postmenopausal women from the EPIC Mediterranean countries (Spain, Greece and Italy, with a high but varied olive oil intake) has also suggested a negative association between olive oil intake and ER− and PR− (progesterone receptor-negative) tumors [50]. However, more recently, a meta-analysis including ten observational studies showed no significant dose–response relationship for olive oil and breast cancer risk, thus highlighting the need for additional prospective studies with better assessment of olive oil intake [51], although conflicting findings between case-control and cohort studies for breast cancer were found [52]. In any case, it should be considered that many epidemiological studies do not distinguish between the consumption of olive oil and EVOO, thus resulting in a great variability in the profile and quality of the oil consumed.

The Mediterranean diet has also been investigated in dietary intervention studies for its cardioprotective effect potential. Some of these trials have discovered secondarily that breast cancer risk was lower in intervention groups. A randomized secondary prevention trial testing the effect of a Mediterranean α-linolenic acid-rich diet showed 61% lower risk of cancer (all subtypes) [53]. In the PREDIMED randomized, nutritional intervention trial, although based on a few incident cases, long-term dietary intervention with EVOO supplementation was associated with 62% reduced breast cancer rates [54]. This trial reported other health benefits of the Mediterranean diet supplemented with EVOO compared to a low-fat control diet, with potential implications in breast cancer risk, such as an effect on body weight and composition [55,56] or antioxidant capacity [57].

Thus, the strongest evidence of the association of the Mediterranean diet and breast cancer risk has been observed in Mediterranean countries [41,54,58], where the consumption of EVOO is higher, but human results remain controversial. The inconsistent association between dietary patterns, foods or nutrients and breast cancer risk is probable due to the complex human diet. Individuals do not have a diet based on isolated nutrients but complex mixtures of components that interact with many biological processes. Thus, several methodological issues and limitations may interfere in human studies, such as the determination of the components of the diet (which is inconsistent among studies), the assessment of the whole diet through questionnaires, geographical variations due to multiple variables (genetics, carcinogenic exposure, culture, or different cancer incidence), or individual characteristics such as hormones, body mass index or exercise practice. Moreover, the potential protective effect of olive oil may strongly depend on its specific composition, i.e., the fatty acid composition and minor compounds, including a great number of polyphenols, as well as the interactions among components. Despite the inconsistent results, epidemiological data suggest a potential beneficial effect of the Mediterranean diet on breast cancer risk, with olive oil playing an important role.

## 3. Effects of Olive Oil on Experimental Mammary Carcinogenesis

Due to the great difficulty in obtaining unbiased data from controlled variables in humans, experimental carcinogenesis become an indispensable tool to obtain mechanistic relationships between dietetic factors and health. Although many models have been developed, the two experimental models more used in mammary carcinogenesis are chemically induced in rodents, specifically the intragastric administration of 7,12 dimethylbenz[α]anthracene (DMBA) and the administration (intravenous, subcutaneous or intraperitoneal) of N-nitrosomethylurea (NMU) [59]. Classical studies reported a stimulating effect of diets high in fat on mammary carcinogenesis. Moreover, for the same amount of fat, a specific effect of the type of lipid has been described [60]. Since the studies carried out by Tannenbaum, several authors have demonstrated the stimulating effect of a diet rich in n-6 PUFA [61] and many of the studies including olive oil have analyzed the influence of this fruit oil in comparison to seed oils rich in n-6 PUFA, such as corn, sunflower or safflower oils. The specific composition of olive oil is a key factor of its potential effect. As already mentioned, EVOO is obtained by physical processes without alterations and with no other treatments, thus being rich in MUFA (oleic acid represents 55–83% of total fatty acids, depending on the oil cultivar, among other variables) but also rich in minor bioactive compounds [11,12,17]. Virgin olive oil, but not the refined one, is the oil that contains all the minor compounds. What is known simply as “olive oil” is not the original product extracted from olives but a blend of virgin and refined oil; therefore, it is not as rich in polyphenols and other minor compounds as EVOO [8,9]. In this sense, few studies have used extra-virgin olive oil in animal studies.

The first investigations with diets containing olive oil, in the NMU-induced carcinogenesis model, showed a stimulating effect of high n-6 PUFA diets (rich in safflower and corn oils) when compared to high olive oil and low-fat diets [62,63]. Moreover, when olive oils with different percentages of oleic acid were tested (54, 70 and 80%), the oil containing the highest oleic acid and the lowest linoleic acid (80% and 5%, respectively) caused histologically more benign adenocarcinomas [64]. In the DMBA-induced rat mammary cancer model, an olive oil diet elicited longer tumor-free time, fewer tumors per rat and lower tumor incidence in comparison to diets high in linoleic acid, what was associated with the low percentage of linoleic acid (18:2n-6) in olive oil [65]. A high-corn-oil diet, but not a high olive oil diet or a high saturated fat diet, also enhanced the growth of pulmonary metastasis [66]. In the MMTV-neu(ndl)-YD5 transgenic mouse model, that develops spontaneous mammary tumors, a 10% marine-derived n-3 PUFA diet was the one that best mitigated breast cancer outcomes, followed by a 10% olive oil diet (with similar effects to saturated fat and plant-derived n-3 diets) and a 10% n-6 PUFA diet showing the poorest outcomes [67].

**Figure 1:**
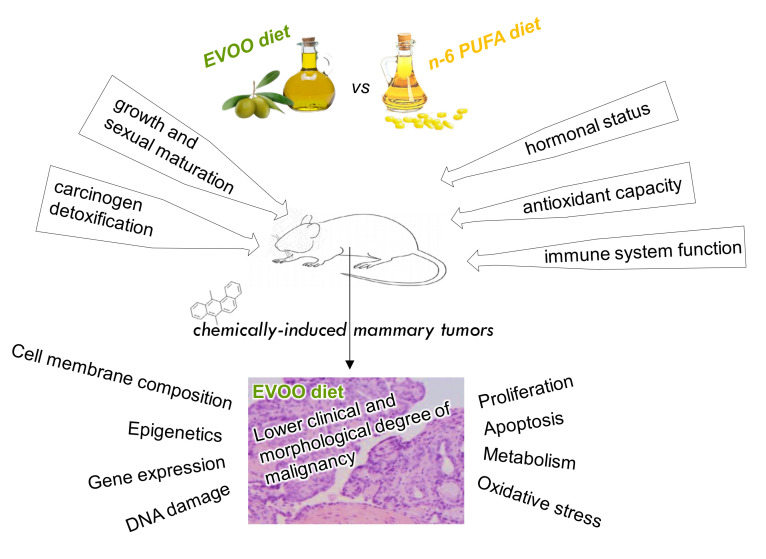
Effects and main mechanisms of action of high-EVOO diets, in comparison to high-seed-oil diets, on experimental mammary carcinogenesis. Animals fed the high-EVOO diet displayed tumors of lower clinical and morphological degree of malignancy. These effects can be related to systemic mechanisms influencing susceptibility and tumor initiation (growth and sexual maturation, liver capacity of carcinogen detoxification, hormone levels, antioxidant capacity and immune function), as well as molecular changes in tumors (in membrane composition, epigenetics, gene expression, DNA damage, oxidative stress, or metabolism, conducting cells to decreased proliferation and increased apoptosis).

Few studies have used extra-virgin olive oil to assess its effects on experimental mammary carcinogenesis. In the NMU model, a normolipidic 4% fat diet showed that animals fed with EVOO had longer latency and lower mortality rates than animals fed sunflower or oleic acid-enriched sunflower oil [68]. Prenatal and prepuberal exposure to moderate quantities of fat (7%) showed the protective effect of an EVOO diet when compared to an n-6 PUFA diet, while high-fat diets (15%) in general had an enhancing effect on DMBA-induced carcinogenesis [69]. On the other hand, in this DMBA-induced model of mammary cancer, a 20% EVOO diet clearly demonstrated a differential effect on the clinical and morphological degree of tumor malignancy in comparison to a 20% corn oil diet, rich in n-6 PUFA. These high-fat diets were administered from weaning or after carcinogen induction, in order to obtain insight into their effects on the initiation and on the promotion of carcinogenesis. In both cases, the high-EVOO diet, in comparison to the high-corn-oil diet, lengthened the tumor latency (interval from carcinogen exposure to palpable appearance of tumor) and decreased tumor incidence (percentage of tumor-bearing animals), multiplicity (number of tumors per animal) and tumor volume [70,71,72,73]. Moreover, these diets also modified the morphological characteristics of tumor aggressiveness. Thus, the high-corn-oil diet, both from weaning and after induction, promoted adenocarcinomas with a high histological degree, more prominent tumor necrosis, stromal invasion and frequent cribriform pattern in comparison with control and high-EVOO diet. Tumors from animals fed the high-EVOO diet displayed a low histopathological grade, with few invasive and necrotic areas [70,74,75,76,77]. Figure 1 depicts the effects of a high-EVOO diet, in comparison to a high-corn-oil diet, on the clinical and morphological manifestation of experimental carcinogenesis, as well as the related mechanisms. Table 2 summarizes the effects and molecular mechanisms of action on olive oil on experimental mammary carcinogenesis

### 3.1. Molecular Mechanisms of the Effects of Olive Oil on Experimental Mammary Carcinogenesis

#### 3.1.1. Effects on Animal Susceptibility and Tumor Initiation

Olive oil has been proposed to have a beneficial effect on breast cancer risk at different systemic levels, including different organs, tissues and body processes beyond the tumoral tissue. One of the earlier processes studied was the hormonal status, since breast cancer is an estrogen-dependent neoplasia (at least at the early steps of carcinogenesis); thus, it is highly influenced by hormones. The cycling nature of hormones and their labile levels make it difficult to draw a relationship, thus data on hormone levels by effect of other fats are inconclusive and few studies have been carried out with olive oil. In pregnant rats, a 7% EVOO diet decreased the estradiol levels in comparison with a corn oil diet and with high-fat diets [69]. However, administration of a 20% EVOO diet from weaning induced no changes in hormones (LH, FSH, estradiol, progesterone, prolactin, insulin and corticosterone) neither in hormone receptors in mammary gland nor in tumors. Such diet increased progesterone receptor in mammary glands at ages around puberty [78].

Growth and sexual maturation are key developmental processes affecting later breast cancer risk [79]. Several studies suggested the effect of dietary fat on such processes. Dietary exposure to a 20% high-corn-oil diet from weaning increased the body weight and mass index of rats, while the isocaloric high-EVOO diet did not modify body weight or mass in relation to a low-fat diet [78,80]. This lack of effect on body weight, despite the great amount of fat in this EVOO diet, was related to the increased level of hypothalamic oxytocin and a nonsignificant increase in plasmatic OEA, both molecules related to body weight regulation [80], and to the regulation of hepatic metabolic genes, such as the uncoupling protein UCP2 [73]. Accordingly, leptin serum levels were lower in animals fed the EVOO diet than in the ones fed the high-n-6-PUFA diet [76]. Concordant results have been observed in sexual maturation, a process that, in humans, is closely related to breast cancer risk [78]. The high-n-6-PUFA diet, but not the high-EVOO diet, advanced morphological sexual maturation in accordance with the increased expression of kisspeptin in the hypothalamus, a marker of puberty. However, little influence of either high-fat diets was observed in the morphology of the mammary gland (number of different epithelial structures) or in molecular differentiation markers (caseins) [78,81].

Recent data have also suggested that the systemic oxidative status may be influenced differentially by these high-fat diets. In healthy rats, oxidative stress-related DNA adducts in liver were increased by effect of a diet supplemented with n-6 PUFA, with no effect by supplementation with olive oil [82]. Although inconsistent results have been obtained, in DMBA-induced animals, high-fat diets seemed to increase oxidative stress, especially the 20% corn oil diet, according to a higher lipid peroxidation in liver [83].

Of special interest is the possible effect of dietary lipids on liver carcinogen detoxification, as they may have a direct impact on cancer initiation. The expression of xenobiotic metabolizing genes (phase I activation of the carcinogen and phase II inactivation of the carcinogen) was studied in the liver and mammary gland of rats fed post-weaning with 20% corn oil or 20% EVOO diets. The results in the liver suggested a balance in favor of the production of active carcinogenic compounds due to the effect of the diet rich in seed oil (increased CYP1A1, CYP1A2 and CYP1B1) and, on the contrary, a greater detoxification due to the effect of the diet rich in EVOO (increased GSTP1, NQO1 and Nrf2 activity), thus modifying the susceptibility to the transformation by environmental carcinogens [72,84,85].

Finally, several studies also pointed at a potential effect of olive oil on the immune system. In DMBA-induced rats, prenatal exposure to a 15% olive oil diet induced changes in spleen production of B and T lymphocytes, as well as higher leucocyte infiltration of tumor [86,87]. Animals fed post-weaning with a 20% EVOO diet showed lower serum levels of proinflammatory cytokines IL1α and leptin than animals fed the isocaloric corn oil diet. In rats fed after induction with this EVOO diet, tumors showed an increase in the infiltration of cytotoxic T lymphocytes (CD8+) [76].

#### 3.1.2. Effects on Tumor Lipid Profile

Dietary lipids may influence the composition, thus the function, of the tumor cell membrane, with an impact on membrane fluidity, lipid peroxidation and signaling transduction pathways mediated by lipids [88]. In DMBA-induced rats, a 20% corn oil diet changed the tumor lipid profile, increasing the relative content of linoleic acid (18:2n-6 PUFA) and decreasing that of oleic acid (18:1n-9 MUFA) in three lipidic fractions, phosphatidylcholine, free fatty acids and triacylglycerides [89].

Other authors have also observed that tumor composition in 18:1n-9 MUFA and 18:2n-6 PUFA reflected the diet, more in the neutral lipid than in the phospholipid fraction [90]. Changes in tumor composition by effect of an olive oil diet have been also observed in N-ethyl-N-nitrosourea-induced tumors [91], in NMU-induced tumors [92] and in spontaneous mammary tumors developed in the MMTV-neu(ndl)-YD5 mouse model [67].

#### 3.1.3. Effects on Tumor Gene Expression

Different cellular processes can be modulated by changes in the gene expression profile. Dietary factors may influence gene expression by a direct effect, but gene modulation can also be the consequence of the interplay among different mechanisms, e.g., cell membrane changes, activation of signaling pathways, or changes in epigenetics. In any case, several studies reported changes, by the effect of olive oil, in tumor genes with a potential role in the carcinogenic process, such as proliferation, differentiation, apoptosis, or metabolism [71,76,93,94].

Transcriptomic analyses demonstrated that a 20% high-EVOO diet induced changes in the mammary gland and tumors. In the mammary gland, especially after a short dietary intervention, a high-corn-oil diet downregulated genes related to the immune system and apoptosis, while a high-EVOO diet modified genes mainly related to metabolism [73]. In DMBA-induced mammary tumors, these high-EVOO diets also mainly modified metabolic genes, while the high-n-6-PUFA diet decreased genes with a role in apoptosis and immune system. Further validation confirmed the downregulation, by the EVOO diet, of the proliferation genes such as Smad1 or Jack2, metabolic genes such as Scd and lower Arg1 and Tngβ1 [76,93]. Other studies on this same model showed changes in the gene expression of proteins involved in important proliferating pathways. The 20% EVOO diet also modulated the mRNA variants of c-erbB1 (coding full-length or truncated EGFR) [71] and Igf2 expression [94].

Prenatal exposure to different 23.4% high-fat diets also resulted in changes on DMBA-induced cancer susceptibility and in the transcriptomic profile of the mammary gland, depending on the type of dietary lipid. The high-olive-oil diet modulated adhesion genes such as Cadm4 or genes related to the immune system (Btn1a1) [95].

#### 3.1.4. Effects on Tumor Epigenetic Mechanisms

EVOO has also shown an effect on epigenetic mechanisms in animal models. A 20% EVOO diet administered from weaning influenced DNA methylation and histone modification in the mammary gland and DMBA-induced tumors. Such diet increased global DNA methylation in mammary glands at ages around puberty, which is in accordance with a decrease in the susceptibility of this gland to mammary transformation. Moreover, global DNA methylation was also increased by the effect of the EVOO diet on mammary tumors, which is in agreement with a lower degree of malignancy. In addition, a 20% high-n-6-PUFA diet, but not the high-EVOO diet, increased the activity of the DNA methyl transferase enzyme, concomitantly with an increase in specific gene methylation of tumor suppressors Rassf1a and Timp3. On the other hand, both high-fat diets had an influence on histone modifications, with the EVOO one decreasing the methylation of histone 4 (H4K20me3) in tumor, while the high-n-6-PUFA diet decreased the methylation of histone 3 (H3K27me3) in the mammary gland, both modifications being associated to increased carcinogenesis. This effect on histone methylation was postulated to be related to the weak tumor-enhancing effect of the high-EVOO diet, in comparison with a low-fat diet, by virtue of the fact of being high in fat [96,97].

#### 3.1.5. Effects on Tumor Proliferation and Apoptosis Pathways

As already mentioned, changes in signaling pathways may be the result of the interplay (as a cause or a consequence) of changes in membrane composition, in gene expression or in epigenetic mechanisms. Some of the pathways more related to the carcinogenesis process are those related to proliferation, apoptosis or metabolism. ErB/Ras is considered a key signaling pathway for growth and proliferation, with relevance in breast cancer. In DMBA-induced rats exposed, post-induction, to high-fat diets, the protein levels and activity of the ErbB1, ErbB2 and ErbB3 were not altered, but a high-EVOO diet decreased the expression of a truncated form of ErbB4. The EVOO diet also induced an increase in p21Ras protein, but a significant decrease in its activity, as well as a decrease in the expression and activity of AKT [75]. Further analyses have also suggested an effect of EVOO on other proliferation pathways, as shown by the decrease in activated Smad [76]. These pathways may be closely related to apoptosis; in fact, a high-EVOO diet increased the levels of activated Caspase-3, which is considered the most important executioner caspase in both intrinsic and extrinsic pathways [75,76]. In mammary glands, the high-corn-oil diet decreased the number of apoptotic cells in different epithelial structures around puberty, while no effect by the isocaloric high-EVOO diet was observed [73].

In the same line, prenatal exposure to a 7% or 15% olive oil diet resulted in a higher apoptotic index of DMBA-induced tumors [87]. Molecular analyses indicated lower levels of anti-apoptotic Bcl2 and higher levels of pro-apoptotic Bak and Caspase-3 activity, compared to tumors from animals fed the corn oil diet [69].

#### 3.1.6. Effects on Tumor Metabolism

One of the hallmarks of tumor cells is metabolic reprogramming [98]. In the DMBA model, a transcriptomic analysis resulted in the modulation of metabolic genes by effect of a high-EVOO diet [76]. At protein and activity levels, this diet, in comparison to a high-corn-oil one, induced modifications, suggesting an increase in glucose uptake and glycolysis (higher levels of Glut1 and PFKL), pentose phosphate pathway (increased GAPDH and PGD), tricarboxylic acid cycle (Citrate synthase and IDH) and energy dissipation by UCP2 [99]. The fact that these changes, that have been associated with tumor aggressiveness, were found in tumors with a lower degree of malignancy, points out that the relevance of metabolic changes probably depends on the interplay with other signaling pathways and processes, such as apoptosis or oxidative stress.

#### 3.1.7. Effects on Tumor Oxidative Stress

Deregulated redox balance and signaling are common hallmarks of carcinogenesis and the potential antioxidant effect of nutrients has raised attention. Although EVOO components have been extensively investigated in vitro, few studies in experimental mammary tumors have addressed the role of EVOO in oxidative stress. In DMBA-induced tumors, high-fat diets increased the markers of oxidative stress. In mammary glands, 20% high-fat diets transitorily decreased the expression and activity of antioxidant enzymes (SOD and Catalase) in comparison with a low-fat diet. The marker of oxidative stress GSSG/GSH (oxidized/reduced glutathione) was increased especially in animals fed the high-n-6-PUFA diet. In mammary tumors, high-fat diets increased the oxidized glutathione (GSSG), whereas the reduced glutathione (GSH) was increased only in the group fed the high-EVOO diet. Although the results were heterogenous, the markers for lipidic oxidative stress (lipofuscine in liver) and DNA damage (8-oxo-dG) suggested a higher oxidative damage by effect of the diet rich in n-6 PUFA [83].

#### 3.1.8. Effects on Angiogenesis and Metastasis

Few studies have focused on the role of olive oil in angiogenesis or metastasis. In NMU-induced mammary tumors, normolipidic diets with 4% of fat from EVOO, refined sunflower oil (rich in n-6 PUFA) and refined sunflower oil enriched with oleic acid had different effects on the renin–angiotensin system. Beyond its cardiovascular effects, this system has been related to the promotion of angiogenesis and tumor growth. The n-6 PUFA diet, but not the EVOO diet, increased the activity of renin–angiotensin [100]. On the other hand, in a model of implantation of metastatic mammary adenocarcinoma, pulmonary metastases developed in lungs were significantly higher in animals fed a high-n-6-PUFA diet than those in animals fed high-olive-oil diet [66].

**Table 2:** Effects and mechanisms of action of olive oil on experimental mammary carcinogenesis.

Animal Model	Dietary Intervention	Carcinogenesis	Molecular/Cellular Mechanisms	Ref.
NMU (50 mg/kg body weight at day 50)	Safflower oil (23%, 5%), corn oil (23%, 5%), olive oil (23%, 5%), coconut oil (23%); post- induction.	Promoting effect of high-safflower-oil and high-corn-oil diets (increased incidence and decreased latent period).	Lipid profile	[62,63,92]
NMU (40 mg/kg body weight at day 50)	Diets at 20% different varieties of olive oil 54/20, 70/15, 80/5 (% oleic acid/% linoneic acid); post-induction.	Lower degree of morphological malignancy by olive oil (80/5).		[64]
NMU (3 × 50 mg/kg body weight at 50, 80 and 110 days)	Diets of 4% EVOO, 4% sunflower oil, 4% oleic acid-enriched sunflower oil; post-weaning.	Protective effect of olive oil (longer latency period, lowest mortality).		[68]
		Diet of 4% oleic acid-enriched sunflower oil induced the highest tumor volume but the lowest morphological malignancy.	Renin–angiotensin system	[100]
Tumor implantation (2 mm^3^ of metastatic mammary tumor)	Corn oil (23%, 5%), olive oil (20%, 5%), beef tallow (20%); 4 weeks pre-implantation.	Increased metastases in 23% corn oil vs. all others.		[66]
DMBA (65 mg/kg body weight at day 50)	Diets of 20% high-linoleic safflower oil, 20% high-oleic safflower oil, 20% olive oil, 20% linoleic-supplemented olive oil.	Preventive effect of olive oil (longer tumor-free time, fewer tumors per rat and lower tumor incidence).	Lipid profile	[65,90]
DMBA (2 × 2 mg/rat at 5 and 6 weeks of age)	Corn oil (7%, 15%), EVOO (7%, 15%); prenatal and lactation.	Smaller tumors with 7% olive oil diet. Promoting effect of high-fat diets.	Hormones (estradiol), apoptosis (Bcl2, Bak, Casp3)	[69]
DMBA (2 × 10 mg/rat at 5 and 6 weeks of age)	Corn oil (7%, 15%), olive oil (7%, 15%); prenatal and lactation.	Preventive effect of olive oil. Promoting effect of high-fat diets.	Immune function, apoptotic index	[87]
DMBA (2 × of 10 mg/rat)	Low-fat, 15% olive oil.		Spleen cellular components, tumor leukocyte infiltrates, apoptosis	[86]
DMBA (5 mg/rat at day 53)	Diets of 3% low-fat, 20% corn oil, 20% EVOO; post-induction.	EVOO vs. corn oil preventive effect. Low histologic grade, few necrotic and invasive areas.		[74,77]
		EVOO vs. corn oil preventive effect. Higher latency time, lower incidence, multiplicity, volume; lower degree of histopathological malignancy.		[70]
			Gene expression—proliferation genes (EGFR, neu)	[71]
			Gene expression—differentiation genes (igf2, H19, VDUP1)	[94]
			Gene expression—differentiation genes (transferrin, β-actin); ZBP1 protein	[81]
			Proliferation and apoptosis pathways (PCNA, ErbB4, Ras, ERK1/2, AKT, Casp3), DNA damage	[75]
DMBA (5 mg/rat at day 53)	Diets of 3% low-fat, 20% corn oil, 20% EVOO; post-weaning/post-induction.	EVOO vs. corn oil preventive effect. Lower tumor incidence and yield.	Growth and sexual maturation (hypothalamic Kiss1)	[78]
			Body mass (plasma OEA, hypothalamic oxytocin)	[80]
			Transcriptomics in mammary gland (immune system, apoptosis, metabolism genes) Liver metabolism (UCP2)	[73]
		EVOO vs. corn oil preventive effect. Lower tumor incidence, yield, volume; lower degree of histopatho- logical malignancy (degree, stromal reaction, necrosis, mitoses).	Transcriptomics in tumor (proliferation, immune system, apoptosis, metabolism genes)	[76,93]
			Expression of Scd, Pfkl, Sema3A, Jak2, Smad1, Casp3, Arg1, Tgfβ1; serum ILα, leptin; CD8 infiltration	[76]
			Epigenetics: DNA methylation (DNMT, Rassf1A, Timp3), histone modifications	[96,97]
			Metabolism (Glut1, PFKL, GAPDH, CS, IDH, UCP2)	[99]
			Carcinogen detoxification (liver and mammary gland Cyp1A1, Cyp1A2, Cyp1B1, Nqo1, AhR, Nfr2, Gstp1)	[84,85]
			Oxidative stress (GSSG/GSH, lipid oxidation, DNA damage)	[83]
DMBA (10 mg/rat at day 53)	Diets of 3% low-fat, 20% corn oil, 20% EVOO; post-weaning.	EVOO vs. corn oil preventive effect. Promoting effect of high-fat diets.	Carcinogen detoxification (Cyp1A1, Cyp1A2, Cyp1B1, Nqo1, AhR, Nfr2, Gstp1), DMBA metabolites and DNA adducts	[72]
DMBA (20 mg/kg body weight at day 21)	Diets of 23,4% olive oil, 23,4% butterfat, 23,4% safflower oil; prenatal.	High safflower oil increased carcinogenesis.	Gene transcription Cadm4, Bbn1a1	[95]
MMTV-neu(ndl)-YD5 mouse	Diets of 10% safflower oil (SA), 3% menhaden oil + 7% SA, 3% flaxseed oil + 7% SA, 10% olive oil, 10% lard.	Menhaden oil better prevented carcinogenesis; safflower oil was the strongest promoter.	Lipid profile	[67]
N-ethyl-N-nitrosourea (180 mg/kg)	Diets of 4% fish oil, 4% olive oil, 4% maize oil; post-induction.	Protective effect of fish oil.	Lipid profile	[91]

## 4. Effect of Olive Oil Components on Mammary Carcinogenesis in In Vivo and In Vitro Models

Numerous compounds in olive oil have been studied for their potential effect in chemoprevention. These studies have been carried out in vivo and, mainly, in vitro in different cell lines. Studies in vivo have been used to elucidate the potential role of these compounds as adjuvant treatment to potentiate the efficacy of certain chemotherapeutic agents and few data have been published about their potential preventive effect as nutritional supplements. On the other hand, extensive research on the effects of EVOO components has been developed using in vitro models. Several breast cancer cell lines with different biological and molecular characteristics have been established trying to represent such a complex and heterogenous disease. Molecular classification of human breast cancer has been used to refine taxonomy simply based on histological assessment, thus defining different basic molecular types. Luminal A is hormone-receptor positive (estrogen-receptor and/or progesterone-receptor positive, ER+, PR+/−) and HER2 negative (HER−) and expresses low levels of the proliferating marker Ki-67. Luminal B is hormone-receptor positive but can express HER2 and has higher levels of Ki67. Triple-negative/basal-like breast cancer is hormone-receptor negative (ER−, PR−) and HER−. HER2-enriched is hormone-receptor negative (ER−, PR−) and HER+. Moreover, other subtypes include normal-like, claudin-low, or apocrine [101]. Different cell lines resembling such molecular subtypes can be used, such as MCF-7, T47D and SUM185 cell lines representing luminal A characteristics (ER+, PR+/−, HER−); BT-474 and ZR-75 for luminal B (ER+, PR+/−, HER2+); MDA-MB-468 and SUM190 for triple-negative basal (ER−, PR− and HER2−); BT549, MDA-MB-231, Hs578T and SUM1315 for triple-negative claudine-low (ER−, PR− and HER2−); SKBR3 and MDA-MB-453 for HER2-enriched (ER−, PR− and HER2+) [102]. The most commonly used cell lines are MCF-7 as hormone-sensitive (representing luminal A) and MDA-MB231 as a model for metastatic triple-negative breast cancer.

### 4.1. Oleic Acid

As the main component of olive oil, several studies have analyzed, in vitro, the effects of oleic acid, with different results reported depending on the concentration and the specific cell line used. Although some results are contradictory, in breast cancer cells, many investigations have found a stimulating influence on proliferation, migration and invasion through different signaling pathways [103,104,105,106]. In the metastatic MDA-MD-231 cells (triple-negative breast cancer), 10 μM oleate was suggested to bind the GPR40 receptor; activate Src proteins, PI3-K and AKT; and increase Ca^2+^ [104]. Higher doses (100 μM) stimulated migration and Stat5 activation through Src, MMPs, COX-2 and LOXs activity-dependent pathways [106,107]. In MCF-7 cells, oleic acid induced ERK1/2 activation and the AP-1-DNA complex, mediated by the activation of EGFR and Src [105] and increased Ca^2+^ [108]. EGFR pathways were also involved in cell migration both in MCF-7 and MDA-MB-231 cells, in which the activation of FFAR1/FFAR4 (GPR40/GPR120), AKT, PI3K, EGFR and NFκB played a pivotal role [109]. On the contrary, in breast cancer cells lines overexpressing HER2 (BT-474 and SKBR3), 10 μM oleic acid decreased the HER2 expression and synergistically enhanced the anti-proliferative effect of the HER2 inhibitor trastuzumab [110,111].

Metabolic pathways have also been associated with oleic acid effects. High doses of oleic acid decreased the viability of MCF-7, but increased viability and migration in highly metastatic cells, such as MDA-MD-231, via enhanced β-oxidation mediated by AMPK activation [112]. A study on a panel of different breast cancer cell lines reported that, in those cells capable of accumulating triacylglycerol, such as MDA-MB-231, oleic acid increased long-term serum-free survival, suggesting a triacylglycerol–FFA cycle induced by oleate [113]. Some studies also pointed at the heterogeneous effects to be dependent on the cell line, since oleic acid decreased the expression of fatty acid synthase (FAS) in the triple-negative cell line MDA-MB-231, while it increased FAS expression in the triple-positive BT-474 line [114].

### 4.2. Hydroxytyrosol

Hydroxytyrosol and tyrosol are the main phenolic alcohols found in EVOO. Fresh olive oil has small concentrations of these compounds, but they increase during storage process due to the hydrolysis secoiridoids [9]. Hydroxytyrosol (HT) has demonstrated antitumoral activities in vivo and in vitro though different mechanisms. In rats induced with DMBA, treatment for six weeks with 0.5 mg/kg of HT inhibited mammary tumor growth and proliferation rate, as well as modified tumor expression profile, modulating genes related to proliferation, apoptosis and the Wnt signaling pathway (increased the expression of Sfrp4) [115]. This treatment with HT also enhanced the total antioxidant capacity in plasma and decreased DNA damage and protein oxidation, suggesting that its combination with chemotherapeutic drugs can reduce the adverse oxidative effects [116].

On the other hand, in vitro HT has demonstrated to decrease cell viability, inhibit cell proliferation due to cell-cycle arrest in the G0/G1 phase and induce apoptosis in MCF-7 cells [117,118]. The cell-cycle arrest was associated to the decrease in peptidyl-prolyl cis-trans isomerase Pin1, which, in turn, decreased the level of the G1 key protein Cyclin D1 [117].

Several signaling pathways have been related with this anti-proliferative effect. In ER-positive cells (MCF-7), HT exerted a clear inhibition of estrogen-dependent activation of ERK1/2 [119]. In HER2-overexpressing cancer cells (SKBR3 and the modified MCF-7/HER2 line), HT significantly reduced the protein expression of the lipogenic enzyme FAS [120], as well as HER2 protein and activation levels [121]. HT also modulated cancer-associated-fibroblasts by suppressing the Chemokine C-C motif ligand 5 (CCL5), thus inhibiting fibroblast-stimulated MCF-7 cell proliferation [122].

Apoptotic pathways are also stimulated by HT treatment. As mentioned before, diet supplementation with HT in DMBA-induced rats induced transcriptomic changes, leading to the induction of Sfrp4, a negative modulator of the Wnt pathway, with a role in the inhibition of proliferation, while it stimulated apoptosis. In addition to Sfrp4, upregulation of Tnfrsf6, Cdkn2a, Cryab, Cabc1 and Il6st and downregulation of Ier3, JunB, c-Jun, Per2, Ccnl2, Apbb3, Car11 were also observed [115]. In SKBR3 cells, HT bound and activated GPER, stimulating the intrinsic apoptotic pathway. Paradoxically, HT increased ERK1/2, although sustained ERK1/2 activation was suggested to lead apoptosis, as evidenced by the upregulation of pro-apoptotic Bax protein followed by a decrease in anti-apoptotic Bcl-2 expression. Thus, HT would induce the GPER-mediated ERK-dependent mitochondria apoptotic pathway, resulting in the release of Cytochrome C (Cyt C), activation of Caspase-9 and Caspase-3, as well as Poly(ADP-ribose) polymerase-1 (PARP-1) inactivation. This effect was accompanied by upregulation in cell-cycle negative regulators, such as p21 and p53, and a reduction in Cyclin D1 expression [123].

The chemopreventive effect of HT has also been related to its antioxidant effect, although the role of oxidative stress and its modulation by antioxidants in cancer is controversial. Reactive oxygen species (ROS) appear to have a complex double effect, having both tumor promoting and tumor suppressing functions. Increased ROS levels have been associated with cancer initiation, transformation and resistance to chemotherapy, but an increase in ROS generation and/or decreased antioxidant defense may activate different cell death pathways and it is the mechanistic effect of many chemotherapeutics. Several studies in human breast cancer have shown that HT supplementation may alleviate the oxidative impact of chemotherapeutic drugs in patients [116]. In vitro, it has been reported that EVOO polyphenols exerted antioxidant effects and prevented DNA-damage at low concentrations (from 1 to 10 μM), but had opposite effects at concentrations higher than 100 μM [124,125]. It has been suggested that, although polyphenols are considered antioxidants, in common cell media and standard conditions, they may act as pro-oxidants. In fact, HT, at high concentrations, produces extracellular hydrogen peroxide (H_2_O_2_) [125], which would be due to sodium bicarbonate in cell media [126]. Moreover, the sensitivity to HT’s anti-proliferative effect has been inversely correlated with the ability of different cell lines to remove H_2_O_2_ from the culture medium [127]. In any case, in normal breast cells (MCF-10A), HT decreased oxidative stress and oxidative DNA damage [128], thus preventing transformation. In MCF-7 cancer cells, HT exerted an antioxidant effect in hypoxic conditions but not in normoxia, also decreasing the PI3K/AKT/mTOR pathway and HIF-1α [129,130]. However, both in hypoxia and normoxia, high doses of HT upregulated Nrf2 and the mRNA of its target genes GSTA2 and HO-1, coding antioxidant proteins. HO-1 was also upregulated in a Nrf-2-independent way [129]. Moreover, high doses of HT in hypoxic MCF-7 cells putatively acted as an aryl hydrocarbon receptor (AhR) agonist, favoring the induction of the angiogenic genes through a HIF-1α-independent mechanism, thus suggesting that HT’s effects under hypoxic conditions are largely dependent on its concentration [130].

HT has also a role in migration inhibition. In triple-negative breast cancer cells (MDA-MB-231, BT549 and Hs578T), HT inhibited metastatic potential in a dose-dependent manner, decreasing epithelial-to-mesenchymal transition (EMT) and tumor cell migration. Such effects were mediated by the dual inhibition of the Wnt/β-catenin and TGFβ signaling pathways. Thus, HT inhibited SMAD2/3-dependent TGFβ signaling and Wnt/β-catenin signaling by decreasing LRP6 (Low-density lipoprotein receptor-related protein-6, a Wnt coreceptor) and β-catenin. Consequently, HT inhibited cyclin D1 protein expression and EMT markers (SLUG, ZEB1, SNAIL and Vimentin), while increased the epithelial marker ZO-1 [131]. HT has also been proposed to inhibit migration by induction of autophagy in metastatic triple-negative (MDA-MB-231) [132] and ER-positive (MCF-7 and T47D) [133] breast cancer cell lines. HT suppressed HGF-induced migration by reversing the inhibition of autophagy proteins (LC3-II/LC3-I and Beclin-1) and reversing upregulation of p62 [132].

### 4.3. Oleuropein

Secoiridoids are a group of compounds found in the species of Oleaceae plants and they comprise the majority of bioactive polyphenols in olive oil and drupes. Oleuropein (OLE) and its biosynthetic precursor, ligstroside, are the main secoiridoids in EVOO. OLE structure comprises a glycosylated ester of elenolic acid with hydroxytyrosol. Most of the secoiridoid phenolic derivatives in EVOO come from oleuropein and ligstroside [134].

OLE has demonstrated an inhibitory effect on viability, cell cycle, proliferation and migration, as well as promotion of apoptosis, in many breast cancer cell lines. Such activities have been related to different molecules and signaling pathways at different levels, since modulation of epigenetic mechanisms, transcriptome, protein levels and protein activation has been described. In triple-negative MDA-MB-468 and MDA-MB-231 cells, OLE induced cell growth inhibition and S-phase cell-cycle arrest-mediated apoptosis. Transcriptomic analyses showed that OLE upregulated the expression of many apoptosis-involved genes in both cell lines, but especially in MDA-MB-468, and those included caspases (Casp1, Casp14), cell-death receptors (FADD, TNFRSF21) and other pro-apoptotic genes, such as GADD45A, CYCS and BNIP2, among others [135]. OLE has also demonstrated an effect on the expression of microRNAs controlling apoptosis proteins. In MDA-MB-231 and MCF-7 cells, OLE increased the expression of pro-apoptotic genes and tumor suppressor miRNAs (miR-125b, miR-16, miR-34a, p53, p21 and TNFRS10B) and decreased the expression of anti-apoptotic genes and oncomiR (bcl-2, mcl1, miR-221, miR-29a, miR-21 and miR-155) [136,137]. In relation to the potential activity of secoiridoids as epigenetic modulators, in vitro screening in different breast cancer cell lines has revealed that decarboxymethyl oleuropein aglycone had inhibitory effects of mTOR and DNA-methyl-transferase (DNMT) and further in silico analyses suggested that this secoiridoid could act as an ATP-competitive mTOR inhibitor and could block the SAM-dependent methylation activity of DNMTs [138]. OLE has also shown, in MCF-7 cells, to decrease the mRNA of several histone deacetylases (HDAC2, -3 and -4) [139,140].

OLE is probably acting on tumor cells thought the modulation of several signaling pathways and its antitumoral effects may be different, depending on the characteristics of the cancer cell lines. In hormone-sensitive cells (MCF-7), high doses of OLE did not interfere with the regulation of gene expression mediated by the estrogen receptor, but could inhibit the estrogen-mediated activation of ERK1/2, suggesting an inhibition of the transduction pathway involving GPR30 and EGF [119]. In this same MCF-7 cell line, OLE reduced the activity of phosphatase PTP1B [141] and induced apoptosis by the upregulation of pro-apoptotic genes (p53 and Bax) and downregulation of anti-apoptotic Bcl-2 [142]. Despite its antitumoral effect on MCF-7 cells [117], OLE has demonstrated stronger cytotoxicity in triple-negative MDA-MB-231 cells than in luminal MCF-7 cells, also inducing apoptosis via the mitochondrial pathway (increased Bax and Casp3 and decreased Bcl2 and Survivin) [143]. Moreover, OLE inhibited cell proliferation by delaying the cell cycle at the S phase, downregulated nuclear factor kappa beta (NF-kB) and cyclin D1 and upregulated the cyclin-dependent inhibitor p21 [143,144]. In addition, in MDA-MB-231 cells (which express Plasminogen activator inhibitor-1, PAI1), but not in MCF-7 cells, OLE exerted anti-proliferative effects by inhibition of PAI-1, which was accompanied by the activation of Caspase-8 [145]. Furthermore, OLE has also shown a stronger antitumor effect in cells overexpressing HER2 (SKBR3 and MCF-7-derived HER2-overexpressing clone) than in HER2-negative MCF-7, decreasing HER2 cleavage and activation [146], downregulating the expression of FAS [120] and stimulating apoptosis through GPER-mediated intrinsic apoptotic pathway (resulting in increased Bax, p21 and p53 and decreased Bcl2 and cyclin D1) [123]. OLE has also shown to enhance tumor apoptosis induced by chemotherapeutics. In nude mice bearing MDA-MB-231 xenografts, OLE (50 mg/kg), in combination with doxorubicin (2.5 mg/kg), induced apoptosis via the mitochondrial pathway. This combined treatment also downregulated NFkB and its target cyclin D1 and downregulated Bcl2 and Survivin [147].

On the other hand, as already mentioned, polyphenols have caught strong attention due to their antioxidant effect, although the role of oxidative stress in cancer is complex and dual. Despite the antitumoral effect of several natural compounds such as polyphenols having been related to antioxidant activity, they can exert a pro-oxidant effect in cancer cells. Recently, it has been reported, in MDA-MB-231 cells, pro-oxidant activity of OLE, being mitochondrial ROS generation the primary mechanism of its antitumor activity (anti-proliferative and pro-apoptotic). Thus, OLE decreased mitochondrial functionality and membrane potential, increased the levels of intracellular ROS and decreased the activity of ROS scavenging enzymes (decreased SOD2 and Catalase). As a consequence, cell-cycle arrest (decreased Cyclin B2 and Cyclin D1) and activation of apoptosis (increased cleaved Caspase 9 and cleaved PARP-1) could be induced [148]. In the same triple-negative cells, OLE also increased ROS and abrogated NF-kB [144]. In MCF-7 cells, EVOO phenolic extracts, where secoiridoids comprised 83% of the total phenolic compounds, also induced intracellular ROS generation and cell death [149]. In any case, the mechanisms by which OLE can generate H_2_O_2_ in cell cultures has been related to the culture conditions [126]; thus, in in vitro studies on the effect of natural antioxidants on cancer cells, methodological issues cannot be ruled out.

Several pathways have also been associated with OLE’s effect in epithelial-to-mesenchymal transition (EMT) and cell migration. This secoiridoid inhibited migration by the induction of autophagy in different cell lines via the inhibition of LC3-II/LC3-I and Beclin-1 and upmodulation of p63 [132,133]. In MCF-7 cells, OLE remarkably decreased migration via the upregulation of p53 and inhibition of SIRT1 and ZEB1, with the consequent increase in the epithelial marker E-cadherin [150]. OLE further suppressed EMT downregulating MMP-2 and MMP-9 [150].

OLE has also been related to other important hallmarks of cancer, such as immune escape. In MDA-MB-231 cells, it induced the inhibition of viability and migration and modulated the triad miR-194-5p/XIST/PD-L1. miR-194-5p and XIST are non-coding RNAs that have been reported to be able to interact with and repress each other. The microRNA miR-194-5p has been positively associated with carcinogenesis, while its potential target, PD-L1, is considered one the major immune escape mechanisms. In this model, OLE decreased miR-194-5p and PD-L1 and upregulated XIST [151]. On the other hand, OLE has also been found to interfere with the aerobic glycolysis enhanced by tumor cells. In MDA-MB-231 cells, glycolysis stress test conducted by measuring the extracellular acidification rate showed a reduced glycolytic rate by effect of OLE [152].

### 4.4. Oleocanthal

Oleocanthal is a derivate by decarboxylation of the aglycone form of oleuropein. This phenolic compound has attracted much scientific attention due to its anti-inflammatory activities similar to ibuprofen, acting as a non-selective COX inhibitor [153]. Moreover, oleocanthal (OC) demonstrated to be an inhibitor of Met, a membrane tyrosine kinase receptor binding the growth factor HGF [154]. In vivo and in vitro, OC has inhibitory effects on breast carcinogenesis though different mechanisms, such as the modulation of apoptosis and changes in several signaling pathways.

In vivo, intragastric administration with oleocanthal (7.5 mg/kg daily for seven weeks) suppressed the initiation and incidence of mammary carcinogenesis in MMTV-PyVT mice developing spontaneous mammary tumors and in a breast cancer patient-derived xenograft model, concomitantly with transcriptomic changes in tumors and with the downregulation of Myc being a key event [155]. In an orthotopic nude mouse model using the MDA-MB-231/GFP human breast cancer cell line, 5 mg/kg (-)-oleocanthal reduced tumor growth and inhibited tumor activation of c-Met, the proliferation marker Ki-67 and the expression of vessel formation marker CD31 [156]. In similar xenograft models, daily oral treatment with OC (5–10 mg/kg) inhibited tumor growth of triple-negative breast cancer cell xenografts, while prevented the estrogen-dependent growth of BT-474 cells and locoregional recurrence [157,158]. OC treatment increased the tumor expression of epithelial markers (E-cadherin) while decreasing the expression of mesenchymal markers (vimentin), as well as decreasing the activation of Met and HER2 receptors and the serum levels of CA 15-3 human breast cancer marker [157].

In several breast cancer cell lines, OC has also demonstrated inhibitory effects on proliferation, migration, invasion and G1/S cell cycle progression [154,156,159]. The OC effects on MDA-MB-231, MCF-7 and BT-474 breast cancer cells were mediated by the inhibition of HGF-induced c-Met activation and its downstream mitogenic signaling pathways, thus decreasing proliferation and cell survival [156]. In MDA-MB-231 cells, OC inhibited HGF-induced AKT and ERK activation and elicited cell-cycle arrest in G1, with decreased cyclin D1 and CDK6 and increased p21 and p27. Met inhibition also downregulated the Brk/Paxillin/Rac1 signaling pathway, with a role in motility, invasion and EMT. Reduced EMT was observed by the increase in epithelial markers (E-cadherin and ZO-1) while the mesenchymal markers vimentin and β-catenin decreased. The downregulation of HGF–Met also led to cell apoptosis by the activation of Caspase-8 and cleavage of receptor interacting protein (RIP), Caspase-3 and PARP-1 [156]. Moreover, OC is a potent inhibitor of mTOR, inducing apoptosis in cells highly expressing this protein, such as MDA-MB-231 [159]. OC had also an antiangiogenic effect and decreased the expression of CD31, a microvessel density marker, in MCF-7 and MDA-MB-231 cells [154].

In addition to OC’s strong effect on inhibiting Met and its downstream signaling effectors, OC has shown to interfere in estrogen-induced proliferation. In ER+ breast cancer cell lines (MCF-7, BT-474 and T47D), OC inhibited proliferation in cells treated with 17-β-estradiol, decreasing ERα expression in BT-474 cells both in vitro and in vivo [158]. On the other hand, in MCF-7 and MDA-MB-231, but not in non-tumorigenic MCF-10A cells, OC inhibited cell migration concomitantly with a modulation of Ca^2+^ elicited by the downregulation of the channel TRPC6 [160].

### 4.5. Luteolin and Apigenin

Flavonoids are a group of polyphenols extensively found in fruits, vegetables and traditional medicinal plants. Apigenin and its major metabolite luteolin are the most concentrated flavones, a class of flavonoids, found in EVOO. Flavones have demonstrated anticancer effects in vivo and in vitro through cellular mechanisms such as inhibition of cell growth, cell cycle arrest, stimulation of apoptosis, or inhibition of angiogenesis and metastasis. Diet supplementation with 0.01% or 0.05% luteolin significantly reduced tumor burden in nude mice inoculated with MDA-MB-231 cells [139]. In the animal model of BALB/c mice inoculated with mouse mammary tumor cells, diets supplemented with 0.02% luteolin reduced tumor volume and tumors showed higher apoptosis and lower angiogenic activity. Luteolin upmodulated the pro-apoptotic genes p53 and Bax and downmodulated the gene expression of anti-apoptotic Bcl-2 [140]. In the DMBA-induced breast tumor model, luteolin had antitumor effects (alone and synergistically in combination with chemotherapy) [141], suppressed the progestin-stimulating tumor growth and had anti-angiogenic activity [142]. Apigenin had an anti-proliferative and pro-apoptotic effect in xenograft models through the inhibition of HER2 expression, VEGF, RANKL and proteasome activity [161,162].

The antitumor effects of flavonoids in vitro have been associated with the modulation of different pathways. In MCF-7 and MDA-MB-468 cells, apigenin induced G2/M cell-cycle arrest by modulating CDK1/cyclin B1, accompanied by ERK inhibition [163]. Similar effects were induced by luteolin in MDA-MB-231 cells, suppressing proliferation and cell-cycle progression by regulating AKT and p21, consequently eliciting PLK1 inhibition and cycle arrest in the G2/M phase, thus decreasing the expression of cyclin B1, cyclin A, CDK1 and CDK2. When cells were stimulated with EGF, luteolin induced a dose-dependent decrease in EGFR gene expression and downregulated the activation of EGF, AKT, ERK1/2 and p38 [164]. In MCF-7 cells, luteolin had a similar action, inhibiting not only the EGF signaling pathways through EGFR, AKT, ERK1/2 and Stat3, but also IGF- and estrogen-induced proliferation [165]. In cells stimulated with IGF, luteolin inhibited the activation of IGF1R and AKT, but not ERK activity. This inhibitory effect was dependent on ERα expression, which was effectively down-regulated by this flavonoid [166]. Luteolin is not considered to bind the estrogen receptors alpha or beta, but it blocks the estrogenic response, also resulting in the modulation of genes related to the estrogen receptor pathway (NCOR1, GTF2H2, NRAS, TAF9, DDX5, NRIP1, POLR2 A and NCOA3) and cell cycle (CDKN1A, CCNA2, PCNA, PLK1 and CCND1). Such effects may be due to epigenetic mechanisms involving histone H4 acetylation [167]. Moreover, luteolin can act as a direct inhibitor of serine/theonine kinases such as PKC, PI3K, GSK3b, CDKs, VRK1 and TLP2 [168,169]. In unstimulated Hs578T, MDA-MB-231 and MCF-7 cells, both flavones, apigenin and luteolin, have also been reported to inhibit PI3K and PKB/AKT, resulting in increased FOXO3a and upregulation of its target genes p21 and p27 [170]. On the other hand, luteolin and apigenin have also shown anti-proliferative and pro-apoptotic effects interfering with lipogenesis by inhibiting FAS expression and activity [120,171]. Luteolin is also able to inhibit NF-kB activation and its target gene c-Myc, thus downregulating the expression of human telomerase reverse transcriptase (hTERT), which encodes the catalytic subunit of telomerase [172].

In relation to the pro-apoptotic effect of luteolin, evidence indicates that this flavone is able to induce several pathways, such as intrinsic, extrinsic and caspase-independent apoptosis. In MCF-7 cells, luteolin stimulated the extrinsic apoptotic pathway by increasing the expression of the death cell receptor DR5 and Caspase-8 activity. Moreover, the activation of the intrinsic pathway was evidenced by increased Bax/Bcl-2 ratio, Cytochrome C release and Caspase-9 activation. Apoptosis executer Caspase-3 was finally activated by luteolin in a dose-dependent way [173]. Activation of the intrinsic pathway has also been described in vivo [162,164,174] and in different cell lines by effect of both apigenin and luteolin [162,170,175]. Finally, luteolin also induced caspase-independent cell death by nuclear translocation of AIF, which was mediated by ERK and p38 activation [176]. Paradoxically, in MDA-MB-231 cells, EGF treatment activated ERK and p38, which were inhibited by luteolin [164].

It has been proposed that luteolin influences EMT, invasion and metastasis modulating different signaling pathways, such as Wnt/β-catenin, Notch, or Receptors Tyrosine Kinase (RTK). In xenograft in vivo models, luteolin inhibited lung metastases of breast cancer [177] and the expression of EMT molecules Vimentin and Slug in primary tumor tissues [178]. Further analysis in vitro in triple-negative cells showed that luteolin reversed EMT by the downregulation of β-catenin and mesenchymal markers (N-cadherin and Vimentin) and upregulation of epithelial markers (E-cadherin and Claudin) [178]. On the other hand, in the triple-negative SUM-149 cell line, which is enriched in tumor-initiating-cell population (CD44+/CD24-), luteolin inhibited Notch-4, with associated loss in cell viability and mammosphere formation. Luteolin acted as a novel inhibitor of RSK (a family of serine/threonine kinases that is part of the MAPK pathway), blocking YB-1/Notch4 signaling [179]. Both in triple-negative (MDA-MB-231) and hormone-sensitive (MCF-7) cell lines, luteolin also inhibited Notch signaling, downregulating Notch-1, Hes-1, Hey-1, Hey-2, VEGF, MMP-2 and MMP-9 mRNA, as well as decreasing the protein levels of VEGF and metalloproteinases MMP-2 and MMP-9 in MDA-MB-231 cells. The modulation of miRNAs [180] and other epigenetic mechanisms such as changes in histone H3 modifications in MMP-9 gene [181] could account, at least partially, for these effects. Thus, in BT-20, a triple-negative cell line expressing androgen receptors, luteolin inhibited proliferation and metastasis and inactivated the AKT/mTOR signaling pathway and subsequent histone remodeling of the MMP9 promoter region [181]. Luteolin could also act as an AhR ligand and decrease the expression of CXCR4, MMP-2 and MMP-9 in MDA-MB-231 cells and in lung metastasis from a mouse melanoma xenograft model [182]. Other signaling pathways have also been associated with luteolin’s blocking effect on EMT, in vitro and in vivo, by the degradation of YAP/TAZ, two transcriptional activators with key roles in tumor–stromal interactions [183]. In MCF-7 cells, a derivate of luteolin (8-C-β-fucopyranoside) suppressed MMP-9 and IL-8 via the downregulation of the MAPK pathway, which resulted in the suppression of the transcription factor AP-1 and NF-κB signaling pathways [184].

In vivo and in vitro results also support an effect of luteolin on other steps of metastasis, such as angiogenesis and extravasation. In a three-dimensional model of extravasation, consisting of MDA-MB-231 spheroids and immortalized lymph endothelial cell monolayers, both flavonoids luteolin and apigenin suppressed pro-intravasative factors, specifically MMP-1 expression and CYP1A1 activity, thus inhibiting the MMP-1-induced activation of the pro-intravasative factor FAK in lymph endothelial cells. Moreover, luteolin also blocked MMP-1-induced Ca^2+^ signaling in these cells [185]. In vivo luteolin suppressed metastasis of MDA-MB-435 and an MDA-MB-231-derived cell line to the lungs. In vitro, relatively low levels (10 µM) of luteolin significantly inhibited the secretion of VEGF [177]. Luteolin and apigenin also inhibited carcinogenesis and angiogenesis in DMBA-induced progestin-stimulated human xenograft tumors, decreasing VEGF and CD31 markers [161,186,187], decreasing, in addition, the acquisition of stem cell-like properties (decreased CD44 expression, aldehyde dehydrogenase activity and mammosphere formation) [186].

Flavones have also been reported to potentially decrease the immune escape of breast cancer cells. Luteolin and, especially, apigenin avoided immune evasion by inhibiting interferon-γ-induced PD-L1 upregulation in several breast cancer cell lines, through the decrease in STAT1 activation. In co-cultures, apigenin increased the proliferation and interleukin-2 synthesis of Jurkat T-cells, thus potentially increasing the vulnerability of breast cancer cells to T-cells antitumor responses [188]. Moreover, apigenin decreased the TNFα/IL-1α-induced release of chemokines (CCL2, GMCSF, IL-1α and IL-6), which regulate cell infiltrates enabling hallmarks such as growth, immune evasion, angiogenesis and metastasis. Such effect was mediated by the downregulation of IKBKe [189].

A novel screening approach (phage display coupled with second-generation sequencing) found 160 direct targets of apigenin, identifying, as a top candidate, the heterogeneous ribonuclear protein A2 (hnRNPA2), a protein that regulates gene expression, splicing, RNA stability and microRNA processing. In MDA-MB-231 breast cancer cells, apigenin inhibited hnRNPA2 dimerization, thus modulating RNA splicing [190].

Flavonoids have been extensively investigated in combination with chemotherapeutics, demonstrating, in vivo, to increase the efficacy and decrease the toxicity of doxorubicin and cyclophosphamide, through oxidative stress-dependent and -independent mechanisms [191,192]. Luteolin alone induced an increase in superoxide dismutase (SOD), catalase (CAT) and glutathione peroxidase (GPx) in non-tumoral tissues and serum, as well as a decrease in tumor [191,192]. However, no effects of luteolin were observed on SOD and CAT in vitro under hypoxia, but there was a decrease in glycolytic flux without affecting glucose uptake. The activation of anti-oxidant enzymes seemed to have a key role in the luteolin protection of healthy tissues, while its anti-tumor effect was suggested to be independent of anti-oxidant enzymes [191]. Apigenin was also shown, in vitro, to induce apoptosis through DNA damage and oxidative stress in cancer cells but not in normal cells [193]. In vitro models have also demonstrated the utility of luteolin as a chemosensitizer. In different cell lines, luteolin had synergistic inhibitory effects with celecoxib (a selective COX-2 inhibitor), via AKT inactivation, targeting different effectors depending on the hormone receptor status of the cell. In ER+ cells (MCF-7 and MCF-7/HER18), combined treatment induced AKT activation and ERK inhibition, while, in ER− cells (MDA-MB-231 and SKBR), there was AKT and ERK activation [194,195]. In MCF-7 cells, luteolin sensitized cells to tamoxifen and downregulated cyclin E2 [196], while apigenin enhanced cisplatin cytotoxicity through p53-induced apoptosis [197]. In both in vitro and in vivo xenograft tumors, ERα+ breast cancer cells (MCF-7 and T47D) were sensitized to indole-3-carbinol by luteolin. The combined treatment downregulated two targets, ERα and the cyclin-dependent kinase (CDK) 4/6/retinoblastoma (Rb) pathway [198]. On the other hand, in hormone-independent MDA-MB-231 cells, the combination of luteolin with paclitaxel increased apoptosis, activated Caspase-8 and Caspase-3 and increased the expression of cell death receptor Fas due to the blocking of STAT3 [199]. A role as an antioxidant may be at the basis of luteolin’s effects, but, as already mentioned, oxidative stress has a dual role on cancer. In this sense, at low concentrations (10 µM), luteolin attenuated doxorubicin-induced cytotoxicity to MCF-7 cells through a combination of antioxidant activity (resulting in reduced doxorubicin-induced ROS generation) and an increase in the anti-apoptotic protein Bcl-2. On the contrary, at high doses (>30 µM), it decreased cell viability [200].

Finally, it is worth mentioning the potential effect of flavonoids on metabolic reprogramming. As already cited, under hypoxia, luteolin decreased glycolytic flux in MCF-7 and in 4T1 mouse mammary cell lines, decreasing lactate and ATP production, while it had no effect on intracellular glucose and glucose uptake [191]. Other flavonoids have been reported to inhibit glucose metabolism in different cancer cells and synthetic flavonoids downmodulated hexokinase 2 in MCF-7 and MDA-MB-231 lines [201].

### 4.6. Other Minor Compounds

Many other EVOO components have been investigated in relation to a potential protective effect on cancer. Lignans are dimeric structures of two phenylpropane units; (+)-pinoresinol and 1-acetoxypinoresinol are the ones found in EVOO. Both lignans inhibited proliferation, induced apoptosis, blocked HER2 activity and reduced the FAS levels in HER2-overexpressing breast cancer cell lines [120,121,202]. In addition to the already mentioned, in these cell models, other compounds, such as tyrosol and elenolic acid, showed similar effects [121]. Pinoresinol has also shown cytotoxic and anti-proliferative effects on different breast cancer cells [203,204,205]. In relation to the complex effect of oxidative stress on carcinogenesis, pinoresinol may prevent the initiation of cancer, as it diminished ROS levels and DNA damage in non-tumorigenic cells (MCF-10A), while, in tumor cells, which possess higher levels of ROS, after H_2_O_2_ treatment, this lignan enhanced ROS levels [205].

Phenolic acids are also found in EVOO, although many fruits, vegetables and mushrooms are richer in such compounds. Caffeic and gallic acids have demonstrated anti-proliferative and pro-apoptotic effects on MCF-7 cells though gene expression modulation of p53, Mcl-1 and p21 [206]. High doses of caffeic acid had anti-proliferative effects in different cells lines, decreasing IGF-I-R and AKT activation, in addition to decreasing ER and cyclin D1 in hormone-dependent cells [207]. Anti-tumor effects of caffeic acid may be partially due to epigenetic mechanisms, such as the inhibition of DNMT1 activity [208].

Uvaol, erythrodiol, oleanolic acid and maslinic acid are the main triterpenes of EVOO. In MCF-7 cells, erythrodiol, uvaol and olanolic acid showed a dose- and time-dependent inhibition of cell growth and proliferation. Erythrodiol stimulated apoptosis associated with ROS production and DNA damage, whereas uvaol’s and oleanolic acid’s growth-inhibitory effects were related to cell-cycle arrest [209]. In these cells, maslinic acid did not demonstrate anti-proliferative or cycle-blocking effect, but decreased ROS production and DNA damage [209]. In MDA-MB-231 and MCF-10A cells, uvaol and erythrodiol (the only difference between the two being the location of one methyl group) had also different effects on oxidative stress. Both triterpenes acted as antioxidants, decreasing ROS levels in basal conditions and, at high doses (10 µM), also decreasing ROS under H_2_O_2_-induced oxidative stress. However, uvaol protected from DNA damage in both cell lines, whereas erythrodiol had the opposite effect, promoting apoptosis and arresting cell cycle in MCF-10A cells [210]. Similar effects have been reported for oleanolic acid, which had antioxidant effects on MCF-10A cells decreasing ROS levels in both the basal state and H_2_O_2_-induced oxidative stress, while it exerted a pro-oxidant effect on MDA-MB-231 cells [211]. Oleanolic acid is a hydroxyl pentacyclic triterpene acid that was demonstrated to have anticancer effects in many breast cancer cell lines [209,212]. This acid induced the inhibition of proliferation, cell-cycle arrest and apoptosis in ER+ cells through an ERα/Sp1-mediated activation of p53 and p21 expression [212]. It also had a pro-apoptotic action in ER− cells, in which oleanolic acid caused alterations in cholesterol homeostasis, associated with lipid-raft disruption, thus inhibition of survival signaling mediated by these membrane structures. This rapid and specific inhibition was the consequence of the disruption of the signaling complexes by decreasing the levels of the mTOR/FRAP1, RAPTOR and RICTOR, which, in turn, decreased mTOR-complex 1 and -complex 2 activity [213]. Recent experiments have reported an antitumor effect of oleanolic acid both in vitro and in vivo, associated with changes in gene expression profile. Screening analyses of MCF-7 cells identified genes related to the p53-, TNF- and mTOR-signaling pathways to be involved in oleanolic acid’s antitumoral effects [214]. Moreover, multiple derivates of oleanolic acid have been developed targeting several signaling pathways [215].

Other EVOO’s minor compounds have attracted attention for their potential anticancer effects, although data may be scarce or nonconclusive. As an example, in colon carcinogenesis, squalene, an acyclic hydrocarbon, was suggested to inhibit HMG-CoA reductase, resulting in a reduction in farnesyl, thus interfering with membrane location and activation of Ras [216]. Few studies have been carried out in breast cancer cells [217] and, currently, squalene investigations are focused on targeting the synthetic pathways with cholesterol-lowering purposes and on their utility as an adjuvant or in the developing of squalene-based nanomedicines [218,219].

**Figure 2:**
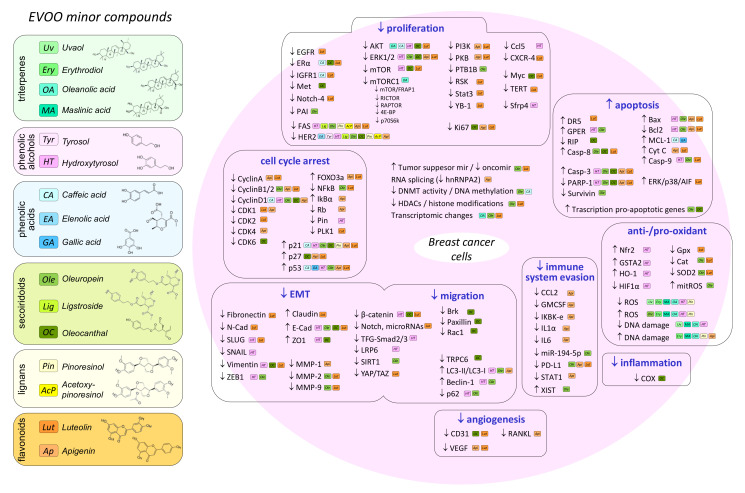
Molecular effects of EVOO minor compounds on mammary carcinogenesis. Compounds are grouped by chemical family and their effects in different molecular targets with a role in the acquisition of tumor hallmarks are indicated. General transcriptomic and epigenetic mechanisms are not associated with a specific hallmark. Many of the molecular targets shown have a role in different tumor hallmarks; in this figure, molecules are classified according to the main effects described in the specific bibliography. ↓, decrease, downmodulation or inactivation; ↑, increase, upmodulation or activation.

Table 3 summarizes the in vivo and in vitro effects of EVOO minor compounds on mammary carcinogenesis. Figure 2 depicts the effects of EVOO minor compounds in molecular targets with a role in the acquisition of tumor hallmarks.

## 5. Concluding Remarks

There is a wealth of knowledge of the potential benefits of EVOO and its minor compounds on health and cancer prevention. The variety of human diet, complexity of interactions among components, individual heterogeneity and multistage process of carcinogenesis make difficult to draw clear conclusions from human studies. Epidemiological data suggest a protective effect of the Mediterranean diet on cancer and, despite the inconsistent results, virgin olive oil seems to play a prominent role. In contrast, extensive in vitro data are reported regarding the anticancer effect of triterpenes, polyphenols (phenolic acids and alcohols, secoiridoids, flavones and lignans) and other EVOO components, with a potential translation to human cancer prevention and treatment. However, caution must be applied, since the effects of such compounds are strongly dependent on methodological issues such as dose, time of exposure and cell type. On the other hand, animal models share, to a large extent, the physiology of nutrition and pathophysiology of the disease with humans and, at the same time, it is possible to segregate the studied parameters. In experimental breast cancer models, dietary lipids modulate clinical and histopathological characteristics of experimental mammary tumors. A diet high in EVOO has a protective effect on carcinogenesis when compared to an isocaloric diet rich in n-6 PUFA, conferring, to tumors, features similar to those induced by a low-fat diet. Although, in general, diets high in fat have a cancer-stimulating influence, evidence points to a preventive effect of EVOO if consumed in moderate quantities. The potential protective effect of EVOO may account for its minor compounds and its ratio of n-3/n-6 PUFAs (higher than the ratios found in many seed oils), while the contribution of MUFA oleic acid is controversial. In vivo and in vitro research highlights that EVOO and/or its compounds can influence the initiation, promotion and progression of carcinogenesis through multiple and varied mechanisms, directly and indirectly, affecting different signaling pathways. The molecular targets of EVOO compounds have a role in the acquisition of cancer hallmarks, resulting in the inhibition of proliferation, cell-cycle arrest, induction of apoptosis, or decrease in several processes, such as migration, immune evasion, angiogenesis and inflammation. Their potential influence on redox balance is considered to have a role in the interplay among different signaling pathways.

In conclusion, EVOO can have a beneficial effect on breast cancer risk, considering a moderate consumption and in the context of Mediterranean diet as a healthy choice from childhood and throughout life. Moreover, although more research is needed on pharmacokinetics, pharmacodynamics, doses, toxicity or effectiveness, evidence highlights the promising potential of several EVOO components as adjuvants in anticancer strategies.

## Figures and Tables

**Table 1:** Olive oil components.

Composition of Olive Oil
Saponifiable Fraction (>98%)
Triacylglycerols and derivatives
16:0 *Palmitic acid*
16:1n-7 Palmitoleic acid
18:0 Stearic acid
18:1n-9 Oleic acid
18:2n-6 Linoleic acid
18:3n-3 Linolenic acid
Unsaponifiable Fraction (<2%)
Non-glyceride esters and waxes
Aliphatic alcohols
Volatile compounds: aldehydes, ketones, alcohols, acids, esters, etc.
Triterpenes: erythrodiol, uvaol, oleanolic acid and maslinic acid
Sterols: β-sitosterol, campesterol, stigmasterol and avenasterol
Hydrocarbons
Squalene
n-alkanes and n-alkenes
Carotenoids: β-carotene and lycopene
Pigments: chlorophylls and pheophytins
Lipophilic phenolics: tocopherols and tocotrienols
Hydrophilic phenolics
Phenolic acids: gallic, vanillic, cinnamic, caffeic, coumanic and elenolic acids
Phenolic alcohols: hydroxytyrosol, tyrosol and their glucosides
Secoiridoids: oleuropein and ligstroside derivates (oleocanthal and oleacein)
Lignans: pinoresinol and acetoxypinoresinol
Flavonoids: luteolin and apigenin

**Table 3:** Overview of the effects of extra-virgin olive oil minor compounds on breast carcinogenesis and associated molecular and cellular mechanisms. Mechanisms induced in combination with chemotherapeutics are not shown.

Component	Model	Carcinogenesis	Molecular/Cellular Mechanisms	Ref.
Triterpenes				
Uvaol	MCF-7	Anti-proliferative	↓ ROS, ↓ H_2_O_2_-induced DNA damage	[209]
	MCF-10A, MDA-MB-231	Decrease in proliferation and survival	↓ ROS, ↓ basal DNA damage (at low doses), ↑ H_2_O_2_-induced DNA damage	[210]
Erythrodiol	MCF-7	Anti-proliferative, pro-apoptotic	↑ ROS	[209]
	MCF-10A, MDA-MB-231	Decrease in proliferation and survival	↓ ROS, ↑ DNA damage; cycle arrest and apoptosis in MCF-10A	[210]
Maslinic acid	MCF-7		↓ ROS, ↓ H_2_O_2_-induced DNA damage	[209]
	MCF-10A, MCF-7, MDA-MB-23	Decrease in proliferation and survival	↓ basal ROS in MCF-10A; ↑ basal ROS in MCF-7 ↑ H_2_O_2_-induced DNA damage	[211]
Oleanolic acid	MCF-7	Anti-proliferative, pro-apoptotic	↓ ROS, ↓ H_2_O_2_-induced DNA damage	[209]
	MCF-10A, MCF-7, MDA-MB-23	Decrease in proliferation and survival	↑ ROS and H_2_O_2_-induced DNA damage in MDA-MB-231 ↓ ROS and H_2_O_2_-induced DNA damage in MCF-10A	[211]
	MCF-7, T47D, SKBR3	Growth inhibition, pro-apoptotic	ERα/Sp1-mediated activation of the p53 gene	[212]
	MCF-7, MDA-MB-231		↓ mTOR-Complex 1 and -Complex2 activity (↓mTOR/FRAP1, RICTOR, RAPTOR, AKT, 4E-BP, p70S6k)	[213]
	MCF-7	Anti-proliferative, pro-apoptotic	Transcriptomic changes; modulation of p53-, TNF- and mTOR-signaling pathways genes ↓THBS1, EDN1, CACNG4, CCN2, AXIN2, BMP4 ↑ATF4, SERPINE1, SESN2, PPARGC1A, EGR1 and JAG1	[214]
Phenolic acids				
Caffeic acid	MCF-7	Decreased viability	↓ p53, ↑ Mcl-1, ↓ p21 (short treatment), ↑ p21 (long treatment)	[206]
	MCF-7, MDA-MB-231	Anti-proliferative, cycle arrest, pro-apoptotic	↓ IGFIR, ↓ AKT activation; ↓ ER, ↓ Cyclin D1 in MCF-7 cells	[207]
	MCF-7, MDA-MB-231		↓ RAR-β methylation	[208]
Elenolic acid	SKBR3, MCF-7/HER2	Anti-proliferative	↓ HER2	[121]
Gallic acid	MCF-7	Decreased viability	↑ p53, ↑ Mcl-1, ↓ p21 (short treatment), ↑ p21 (long treatment)	[206]
Phenolic alcohols				
Tyrosol	SKBR3, MCF-7/HER2	Anti-proliferative	↓ HER2	[121]
Hydroxyty-rosol	In vivo (DMBA)	Growth inhibition, anti- proliferative	Transcriptomic changes in tumors; modulation of apoptosis, cell cycle, proliferation, differentiation, survival and transformation pathways genes; ↑ sfrp4	[115]
			Plasma: ↑ antioxidant capacity, ↓ DNA and protein damage	[116]
	MCF-7	Decreased cell viability, anti-proliferative, blocked G(1)-to-S transition, pro-apoptotic	↓ Pin1, ↓ Cyclin D1	[117,118]
	MCF-7	Anti-proliferative	↓ ERK1/2	[119]
	SKBR3, MCF-7/HER2	Anti-proliferative, pro-apoptotic	↓ FAS, ↓ HER2	[120,121]
	cocultures MCF-7- fibroblast	Inhibition of fibroblast-stimulated MCF-7 proliferation	↓ CCL5 expression in aging fibroblasts	[122]
	SKBR3	Pro-apoptotic	↑ GPER, ↑ ERK1/2, ↑ Bax, ↓ Bcl-2, ↑ Casp-9, ↑ Casp-3, ↓ PARP-1, ↑ p21, ↑ p53, ↓ Cyclin D1	[123]
	MDA and MCF-7	Anti-proliferative, pro-apoptotic	Extracellular production of hydrogen peroxide	[125]
	MCF-10A, MDA-MB-231, MCF-7		Prevents oxidative DNA damage ↓ intracellular ROS level in MCF-10A	[128]
	MCF-10A, MDA-MB-231		Pro-oxidant under specific growth conditions	[126]
	MCF-7		Antioxidant; ↑ Nrf2, ↑ GSTA2, ↑ HO-1	[129]
	MCF-7 under hypoxic conditions		↓ PI3K/AKT/mTOR pathway, ↓ HIF-1α, ↓ PARP-1 At high doses ↑ VEGF, ↑ AM, ↑ Glut1	[130]
	MDA-MB-231, BT549, Hs578T	Inhibition of EMT, migration and metastatic potential	↓ SMAD2/3-dependent TGFβ signaling, ↓ p-LRP6, ↓ LRP6, ↓ β-catenin, ↓ cyclin D1 ↓ SLUG, ↓ ZEB1, ↓ SNAIL, ↓ Vimentin; ↑ ZO-1	[131]
	MCF-7 and T47D	Inhibition of migration and invasion	Induction of autophagy	[133]
	MDA-MB-231	Inhibition of migration and invasion	Induction of autophagy; ↑ LC3-II/LC3-I, ↑ Beclin-1, ↓ p63	[132]
Secoiridoids				
Ligstroside	SKBR3, MCF-7/HER2	Anti-proliferative, pro-apoptotic	↓ FAS, ↓ HER2	[120,121]
Oleuropein	In vivo (cancer- stem-cell-enriched orthotopic model)	Treatment with decarboxymethyl oleuropein reduced carcinogenesis	↓ DNMT, ↓ mTOR	[138]
	MCF-7	Decreased cell viability, inhibited cell proliferation, blocked G(1)-to-S transition, pro-apoptotic		[117]
	MDA-MB-468, MDA-MB-231	Growth inhibition, S-phase cell-cycle arrest-mediated apoptosis	Transcriptomic changes in apoptosis-involved genes (Casp1, Casp14, FADD, TNFRSF21, GADD45A, CYCS and BNIP2)	[135]
	MCF-7, MDA-MB-231	Pro-apoptotic	Increased the expression of pro-apoptotic genes and tumor-suppressor miRNAs; decreased the expression of anti-apoptotic genes and oncomiR	[136]
	MCF-7	Anti-proliferative, pro-apoptotic, inhibition of migration	↓ mir-21, ↓ mir-155	[137]
	MCF-7	Reduced viability and invasiveness, pro-apoptotic	↓ HDAC2, ↓ HDAC3, ↓ HDAC4	[139,140]
	MCF-7	Anti-proliferative	↓ ERK1/2	[119]
	MCF-7	Reduced viability, cell- cycle arrest	↓ PTP1B	[141]
	MCF-7	Pro-apoptotic	↑ p53, ↑ Bax, ↓ Bcl-2	[142]
	MDA-MB-231	Anti-proliferative, pro-apoptotic, cell-cycle arrest	↑ Bax, ↑ Casp3, ↓ Bcl2, ↓ Survivin; ↓ NF-kB, ↓ CycD1, ↑ p21	[143]
	MDA-MB-231	Pro-apoptotic	↑ ROS, ↓ NF-kB	[144]
	MDA-MB-231	Cell growth inhibition	↓ PAI-1, ↑ Casp8	[145]
	SKBR3, MCF-7/HER2		↓ FAS	[120]
	SKBR3, MCF-7/HER2, MCF-10A/HER2	Anti-proliferative, pro-apoptotic	↓ HER2	[121,146]
	SKBR3	Pro-apoptotic	↑ GPER, ↑ Bax, ↓ Bcl-2; ↑ Casp-9, ↑ Casp-3, ↓ PARP-1; ↑ p21, ↑ p53, ↓ Cyclin D1	[123]
	MCF-10A, MDA-MB-231		Pro-oxidant under specific growth conditions	[126]
	MDA-MB-231	Anti-proliferative, pro-apoptotic	Pro-oxidant activity, ↓ SOD2 ↓ catalase, ↑ intracellular and mitochondrial ROS ↓ CycB2, ↓ CycD1, ↑ Casp9, ↓ PARP-1	[148]
	MCF-7 and T47D	Inhibition of migration and invasion	Induction of autophagy	[133]
	MDA-MB-231	Inhibition of migration and invasion	Induction of autophagy; ↑ LC3-II/LC3-I, ↑ Beclin-1, ↓ p62	[132]
	MCF-7	Inhibition of migration	↓ Sirt1, ↑ ECad, ↓ ZEB1, ↓ MMP-2, ↓ MMP-9, ↑ p53	[150]
	MDA-MB-231	Decreased viability and migration	↓ miR-194-5p, ↓ PD-L1, ↑ XIST	[151]
	MDA-MB-231		↓ glycolysis rate	[152]
Oleocanthal	In vivo (MMTV-PyVT; patient-derived xenograft)	Suppressed initiation and incidence	Transcriptomic changes, ↓ Myc	[155]
	In vivo (MDA- MB-231 xenograft)	Inhibition of tumor proliferation and growth	↓ c-Met, ↓ Ki-67, ↓ CD31	[156]
	In vivo (BT-474 and MDA-MB-231 xenografts)	Prevention of locoregional recurrence, tumor growth inhibition	↓ c-Met, ↓ HER2; ↑ ECad, ↓ Vimentin; ↓ CA 15-3 in serum	[157]
	In vivo (BT-474 xenograft)	Tumor growth inhibition	↓ ERα	[158]
	MCF-7, BT-474, MDA-MB-231	Inhibition of proliferation and survival	↓ Met, ↓ AKT, ↓ ERK; ↓ CycD1, ↓ Cdk6, ↑ p21, ↑ p27; ↓ Brk/Paxillin/Rac1; ↑ ECad, ↑ ZO-1, ↓ Vimentin, ↓ β-catenin; ↑ Casp8, ↑ Casp3, ↓ RIP, ↓ PARP-1	[156]
	MCF-7, MDA-MB-231	Anti-proliferative, inhibition of migration and invasion	↓ c-Met activation. ↓ microvessel density marker (CD31)	[154]
	MCF-7, T47D, MDA-MB-231	Anti-proliferative	↓ mTOR and inducing apoptosis in MDA-MB-231 cells	[159]
	MCF-7, BT-474, T47D	Inhibition of estrogen- induced proliferation	↓ ERα in BT-474	[158]
	MCF-7, MDA-MB-231	Anti-proliferative, inhibition of migration	Modulation of Ca^2+^ entry through TRPC6	[160]
Lignans				
Pinoresinol	SKBR3, MCF-7/HER2		↓ FAS	[120]
	SKBR3, MCF-7/HER2, MCF-10A/HER2	Anti-proliferative, pro-apoptotic	↓ HER2	[121,202]
	MCF-7 and TD47D	Cytotoxicity		[203]
	MDA-MB-231	Anti-proliferation	↑ p21	[204]
	MDA-MB-231, MCF-7, MCF-10A	Cytotoxic, anti-proliferative and pro-oxidant	↓ ROS, ↓ DNA damage in MCF-10A cells; ↑ ROS in cancer cells after H_2_O_2_ treatment	[205]
Acetoxypinoresinol	SKBR3, MCF-7/HER2		↓ FAS	[120]
	SKBR3, MCF-7/HER2, MCF-10A/HER2	Anti-proliferative, pro-apoptotic	↓ HER2	[121,202]
Flavonoids				
Apigenin	In vivo (BT-474 xenograft model)	Tumor growth inhibition, anti-proliferative, pro- apoptotic	↓ Ki-67, ↓ HER2, ↓ VEGF, ↑ RANKL	[161]
	In vivo (MDA- MB-231 xenograft)	Tumor growth inhibition, pro-apoptotic	↑ ubiquitinated proteins, ↑ Bax, ↑ IκBα	[162]
	Hs578T, MDA- MB-231, MCF-7	Anti-proliferative, cell- cycle arrest, pro-apoptotic	↓ PI3K, ↓ PKB, ↑ FOXO3a, ↑ p21, ↑ p27; ↑ p53; ↑ PARP-1; ↑ Cyt C	[170]
	SKBR3, MCF-7/HER2		↓ FAS	[120]
	MDA-MB-231	Anti-proliferative, pro-apoptotic	↑ Casp-3, ↑ proteosome activity, ↑ ubiquitinated proteins, ↑ Bax, ↑ IκBα, ↑ PARP-1	[162]
	MCF-7, MDA-MB-468	Growth inhibition, cycle arrest	↓ cyclin B1, ↓ cyclin D1, ↓ cyclin A, ↓ CDK1, ↓ CDK4, ↓ Rb (in MCF-7), ↓ ERK (in MDA-MB-468)	[163]
	T47D, MDA-MB-231	Anti-proliferative, pro-apoptotic	↑ Casp3, ↓ PARP-1, ↑ Bax ↓ Bcl-2; ↑ LC3-II	[156]
	MDA-MB-231 spheroids—lymph endothelial cells		↓ MMP-1, ↓ CYP1A1 in MDA-MB-231 cells ↓ FAK in lymph endothelial cells	[185]
	MDA-MB-468, SKBR3, mouse 4T1 cells		↓ PD-L1, ↓ STAT1 activation, ↑ T-cell proliferation in co-culture	[188]
	MDA-MB-231	Decreased viability	↓ CCL2, ↓ GMCSF, ↓ IL-1α, ↓ IL-6, ↓ IKBK-e	[189]
	human breast tumor phage display cDNA librar; MDA-MB-231		160 direct targets, hnRNPA2 top candidate ↓ hnRNPA2 activation, modulation of splicing in MDA-MB-231 cells	[190]
	MCF-7, MDA MB-231	Decreased viability, pro-apoptotic	↑ lipid peroxidation, ↑ DNA damage	[193]
Luteolin	In vivo (MDA-MB-231 xenograft)	Reduced tumor burden Reduced tumor growth	↓ Ki-67	[164,180]
	In vivo (mouse mammary tumor cells)	Tumor growth inhibition, pro-apoptotic, angiogene- sis inhibition	↑ p53, ↑ Bax, ↓ Bcl-2	[174]
	In vivo (DMBA- induced)	Tumor growth inhibition	Antioxidant, ↑ SOD, ↑ CAT, ↑ GPx	[192]
	In vivo (DMBA- induced)	Tumor growth inhibition, anti-angiogenic	↓ VEGF, ↓ CD31	[187]
	In vivo (T47D xenograft)	Tumor growth inhibition, anti-angiogenic	↓ VEGF, ↓ CD31	[186]
	In vivo (MDA-MB-435, MDA-MB-231(4175)LM2 xenograft)	Inhibition of lung metastases		[177]
	In vivo (MDA-MB-231 xenograft)	Inhibition of lung metastases	↓ Slug, ↓ Vimentin	[178]
	In vivo (4T1 implantation)	Tumor growth inhibition	↓ YAP, ↓ TAZ	[183]
	In vivo (MCF-7, 4T1 implantation)		↑ SOD, CAT in serum ↓ SOD, ↓ CAT in tumor	[191]
	MDA-MB-231	Cell growth inhibition, cell-cycle arrest, pro-apoptotic	↑ p21, ↓ PLK1, ↓CycB1, ↓ CycA, ↓ CDK1, ↓ CDK2; ↑ Bax; ↓ EGFR, ↓ AKT, ↓ ERK1/2, ↓ p38	[164]
	MCF-7	Anti-proliferative, cell- cycle arrest, pro-apoptotic	↓ EGFR, ↓ AKT, ↓ ERK1/2, ↓ Stat3	[165]
			↓ IGFR1, ↓ AKT, ↓ ERα	[166]
	MCF-7	Anti-proliferative	Regulation of gene expression (estrogen receptor pathway and cell cycle genes)	[167]
	Hs578T, MDA- MB-231, MCF-7	Anti-proliferative, cell- cycle arrest, pro-apoptotic	↓ PI3K, ↓ PKB, ↑ FOXO3a, ↑ p21, ↑ p27; ↑ p53; ↑ PARP-1; ↑ Cyt C	[170]
	MDA-MB-231	Anti-proliferative, pro-apoptotic	↓ FAS	[171]
	SKBR3, MCF-7/HER2		↓ FAS	[120]
	MDA-MB-231	Anti-proliferative, cell- cycle arrest, pro-apoptotic	↓ NF-kB, ↓ Myc, ↓TERT	[172]
	MCF-7	Anti-proliferative, cell- cycle arrest, pro-apoptotic	↑ DR5, ↑ Casp8, ↑ Bax, ↓ Bcl-2, ↑ Casp9, ↑ Casp3	[173]
	MCF-7, MDA- MB-231, SKBR3	Decreased viability, pro-apoptotic	↑ ERK/p38 activation, AIF translocation	[176]
	MDA-MB-435, MDA-MB-231(4175)LM2	Anti-proliferative, pro-apoptotic, reduced migration	↓ VEGF secretion	[177]
	MDA-MB-231, BT5-4	Inhibition of migration and invasion	↓ β-catenin, ↓ N-cadherin, ↓Vimentin, ↑ E-cadherin,	[178]
			↑ Claudin	
	SUM-149	Reduced enrichment in stem cells and growth, pro-apoptotic	↓ RSK, ↓ YB-1, ↓ Notch4	[179]
	MCF-7, MDA-MB-231	Anti-proliferative, cycle arrest, decreased migration	↓ Notch1, ↓ Hes1, ↓ Hey1, ↓ Hey2, ↓ VEGF, ↓ MMP-2, ↓ MMP-9 ↑ miR-34a, ↑ miR-181a, ↑ miR-139-5p, ↑ miR-224, ↑ miR-246, ↓ miR-155	[180]
	BT-20	Anti-proliferative, reduced migration and invasion	↓ AKT, ↓ mTOR, ↓ MMP-9, ↓ H3K27ac, ↓ H3K56ac	[181]
	MDA-MB-231	Reduced viability, cycle arrest, pro-apoptotic	↓ CXCR4, ↓ MMP-2, ↓ MMP-9	[182]
	MDA-MB-231, 4T1	Reduced viability, inhibition of colony formation	↓ YAP, ↓ TAZ, ↓ N-Cad, ↓ Vimentin, ↓ FN1, ↑ E-Cad	[183]
	MDA-MB-231 spheroids—lymph endothelial cells		↓ MMP-1, ↓ CYP1A1 in MDA-MB-231 cells ↓ FAK, ↓ Ca^2+^ release in lymph endothelial cells	[185]
	T47D, BT-474	Reduced viability, pro-apoptotic	↓ VEGF	[186]
		Reduced mammosphere formation in T47D cells		
	MDA-MB-468		↓ PD-L1	[188]
	MCF-7, 4T1		↓ glycolytic flux (under hypoxia)	[191]

↓: decrease, downmodulation or inactivation; ↑: increase, upmodulation or activation.
